# Follicular Dendritic Cell Sarcoma in Mediastinum: A Case Study and Literature Review

**DOI:** 10.1111/crj.70140

**Published:** 2025-11-21

**Authors:** Qingxia Xu, Chong Zhang, Yang Ma, Longquan Xiang

**Affiliations:** ^1^ Department of Pathology Jining No. 1 People's Hospital Jining Shandong China; ^2^ Department of Respiratory Medicine Decheng District Hospital of Traditional Chinese Medicine Dezhou China

**Keywords:** diagnosis, follicular dendritic cell sarcoma, immunohistochemistry, mediastinum

## Abstract

Follicular dendritic cell sarcoma (FDCS) is an uncommon malignant neoplasm that arises from follicular dendritic cells (FDCs). The mediastinum is a more unusual site of FDCS. In this document, we detail a case involving the complete surgical removal of FDCS located in the mediastinum. A 28‐year‐old woman presented with symptoms of right chest pain. Accompanying symptoms include chest tightness, shortness of breath, and faintness. Chest computed tomography was performed and revealed abnormal enhancement in the mediastinal region. An excisional biopsy was carried out, and through the aid of immunohistochemistry (IHC), a diagnosis of FDCS was confirmed. Following surgery, the patient underwent radiotherapy for 27 sessions. The patient was followed up by the oncology service for 6 years and was still alive at the time of drafting this report. This exceedingly uncommon case underscores the challenges in making a differential diagnosis and emphasizes the significance of diagnostic indicators, including histopathology and IHC, in establishing a diagnosis. Clinicians should be alert to the possibility of encountering this disease and take into consideration various characteristics to avoid misdiagnosis.

## Graphical abstract

Follicular dendritic cell sarcoma (FDCS) is a rare malignant tumor that originates from follicular dendritic cells. We report a case of a completely resected FDCS arising in the mediastinum. This extremely rare case highlights the difficulties in differential diagnosis and the importance of diagnostic clues as histopathology and immunohistochemistry in the establishment of a diagnosis.

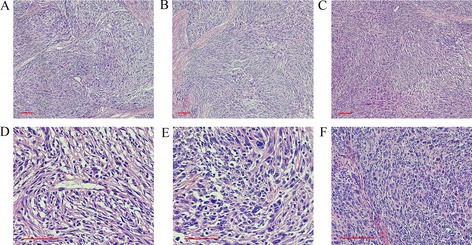

## 1 Introduction

Follicular dendritic cell sarcoma (FDCS) was initially documented in four cases of primary lymph node malignancy by Monda et al. in 1986 [1, 2]. FDCS is an uncommon malignant tumor that arises from follicular dendritic cells (FDCs). FDCS predominantly occurs in lymph nodes located in the head‐and‐neck region [3]. The tonsils and abdominal organs are the most frequent extranodal areas involved [4, 5]. Yet the mediastinum is a rare site of FDCS. The atypical clinical characteristics coupled with varied morphologic features increase the difficulty in reaching an accurate diagnosis of FDCS. Herein, we present a case report of a mediastinal FDCS that was completely resected.

## 2 Case Study

A 28‐year‐old female presented to the thoracic surgery department at the hospital with a chief complaint of right‐chest pain persisting for the past year. The pain was gradually progressive, and the chest pain broke out violently once in this process. Accompanying symptoms include chest tightness, shortness of breath, and faintness. The patient has consented to the publication of this case.

**FIGURE 1:**
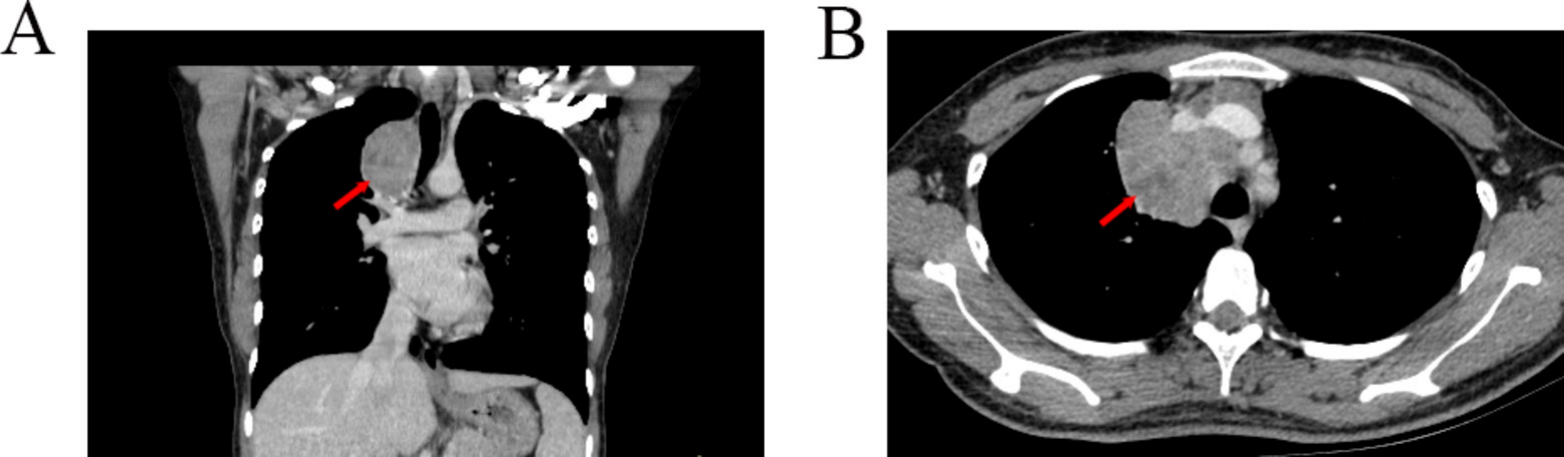
Computed tomography scan revealing a mediastinal mass that compresses the surrounding tissue.

The chest computed tomography (CT) scan revealed a large occupying lesion situated in the mediastinum, exerting compression on the anterior‐right tracheal prominence (Figure 1). There were no abnormalities in the hematological examination. A CT‐guided needle biopsy was performed before the major surgery. Hematoxylin–eosin staining showed spindle‐shaped tumor cells with a varied staggered pattern. It was difficult to make a definitive diagnosis based on imaging examination and puncture biopsy.

**FIGURE 2:**
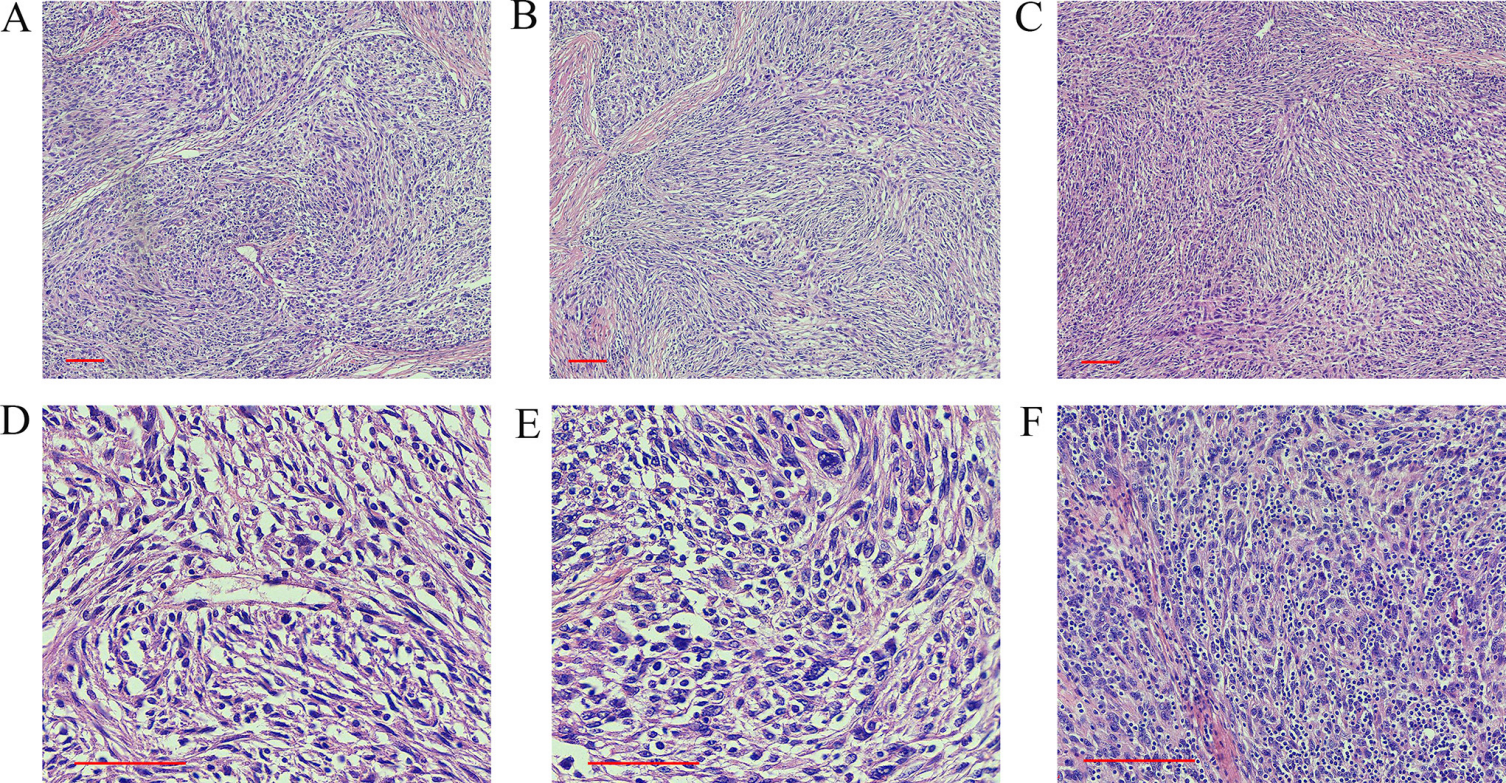
Histological examination of the follicular dendritic cell sarcoma in the mediastinum. HE staining in different views. Scale bars: 60 μm.

After the evaluation of the preoperative examination, we considered that the mass expressed the characteristics of malignancy and compressed the surrounding tissue in an invasive growth pattern. Excision biopsy of the tumor was done and showed a well‐circumscribed and encapsulated tumor. Macroscopically, the size of the mass was measured to be about 6.5 × 5.5 × 4.5 cm. The cut surface of the mass appeared gray white with a fine texture. Microscopically, FDCS is distinguished by the growth of spindle‐shaped cells that arrange themselves into bundles and swirls. Tumor cells display a storiform or palisading arrangement, with the stroma possessing a myxoid feature. The nuclei are predominantly oval shaped, exhibiting a vesicular chromatin pattern, featuring small nucleoli and limited mitotic activity. A distinctive characteristic is the scattered distribution of small lymphocytes among the tumor cells, giving it a thymoma‐like appearance. Pseudonuclear inclusions and multinucleated giant cells may be present in some microscopic views. Cellular atypia and coagulative necrosis were observed (Figure 2).

**FIGURE 3:**
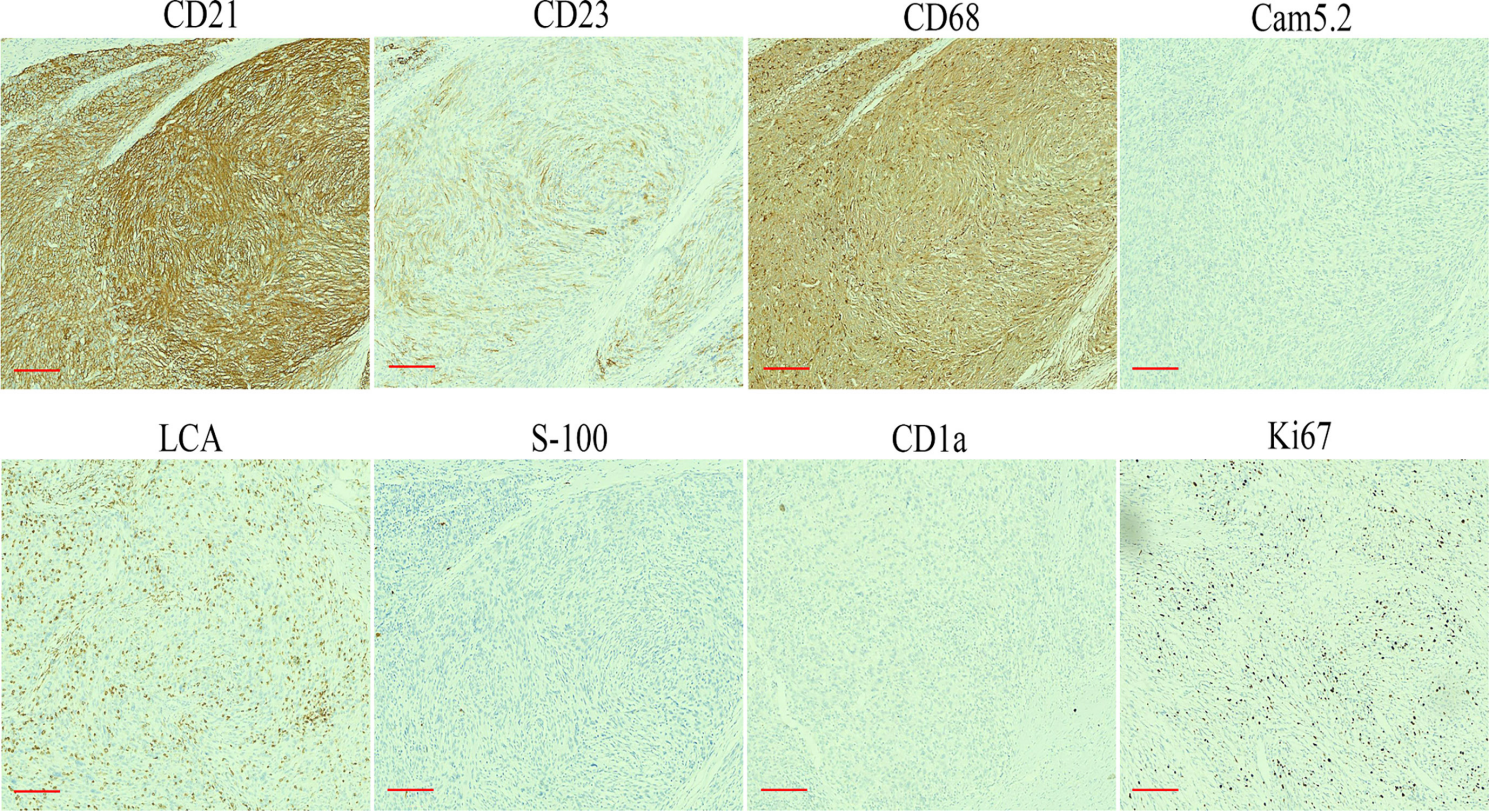
Immunohistochemical characteristics of the follicular dendritic cell sarcoma in the mediastinum. Immunohistochemistry shows that the tumor cells are positive for CD21, CD68, and CD23 staining and negative for Cam5.2, CD1a, S‐100, and LCA staining. The Ki67 proliferation index is approximately 10%. Scale bars: 60 μm.

The IHC characteristics match those of FDCs. Particularly, markers such as CD21, CD23, and CD35 are valuable for their identification. The tumor cells were positive for CD21, CD68, and CD23, and not reactive for Cam5.2, LCA, CD1a, and S‐100. The Ki67 proliferation index was approximately 10% (Figure 3). Based on the hematoxylin–eosin staining and IHC findings, the diagnosis was confirmed to be FDCS. The patient received adjuvant postoperative radiotherapy 27 times. Six years after surgery, follow‐up examinations revealed no signs of recurrence.

## 3 Discussion

FDCS is a rare sarcoma of low to intermediate grade, and about 300 cases were reported in the English literature [3, 6]. The most frequent site for these tumors was within the lymph nodes; other common sites were the painless cervical and extranodal organs including the liver, head and neck, spleen, lungs, pancreas, or bowel [7, 8, 9]. The mediastinum is a rare origin site among the cases [7].

The etiology for FDCS is not clear at present. Exposure to the Epstein–Barr virus (EBV) was reported to be involved in the pathogenesis of FDCS [10]. This kind of FDCS is named inflammatory pseudotumor‐like FDCS for having different clinical and pathologic features. The markers of CD21 and EBV are instrumental in diagnosing FDCS [11]. Another research proposed that hyaline vascular type Castleman's disease (HVCD) might be a precancerous lesion of FDCS [12]. Cloning amplification of FDCs in HVCD might be the related mechanism [13, 14].

The clinical feature of FDCS is generally nonspecific, which might lead to a misdiagnosis initially. The majority of tumors appear as slowly growing painless masses with clear macroscopic boundaries [15]. Some types of FDCS develop symptoms only when they are large enough to compress surrounding organs. Imaging diagnosis plays a crucial role in the evaluation of soft tissue sarcomas. CT scanning can be utilized to assess the size, morphology, density, and involvement of surrounding structures of the tumor. FDCS may appear as single or multiple indistinct soft tissue masses on CT images, with heterogeneous density and intermingling with surrounding structures. This heterogeneous density may reflect the tumor's internal heterogeneity, possibly due to bleeding, necrosis, or varying degrees of cellular proliferation. In this case, the patient's enhanced CT scan revealed an irregular soft tissue density shadow in the right upper anterior mediastinum, with significant uneven enhancement on the enhanced CT scan. This pronounced enhancement may be associated with the tumor's rich vascularity or metabolic activity of tumor cells.

Microscopically, the tumor cells are organized in spindle‐shaped bundles with varying numbers of lymphocytes infiltrating the stroma. The differential diagnosis of FDCS mainly involves tumors/sarcomas of various types of dendritic cells and histiocytic origin, which share similar histological features, posing challenges in differentiation. Additionally, many tumors such as sarcomatoid carcinoma, poorly differentiated squamous cell carcinoma, and malignant melanoma can exhibit analogical microscopic morphology. Therefore, making a precise diagnosis of FDCS based solely on morphology can be challenging. Immunohistochemistry is critical for confirming the diagnosis. Histiocytic sarcoma tumor cells display histiocytic features, arranged in sheets, with poor adhesion, and appear round or oval, often showing multinucleation. Immunohistochemical markers CD68, CD123, CD163, and Lysozyme are positive. In Langerhans cell histiocytosis/Langerhans cell sarcoma, tumor cells are oval shaped with folded, indented, lobulated nuclei, often exhibiting nuclear grooves, which are key diagnostic features. Immunohistochemical markers CD1α, Langerin, and S‐100 are positive. Dendritic cell sarcoma tumor cells range from spindle to oval shaped, arranged in clusters or whirls, often mixed with a lymphocyte background, making it difficult to distinguish from FDCS. Immunohistochemical expression includes S‐100, vimentin, and fascin. Inflammatory myofibroblastic tumors consist of myofibroblastic proliferation with a lymphoplasmacytic background, lacking the characteristic fascicular and whorled arrangement, with less prominent cellular atypia. Immunohistochemical markers MSA, SMA, and h‐caldesmon are positive. Malignant melanoma tumor cells exhibit diverse morphologies, appearing spindle shaped or epithelioid, with clear cell borders and prominent nucleoli. Immunohistochemical markers HMB‐45, Melan‐A, and S‐100 are positive. FDCS originates from FDCs, which mainly exist in lymphoid follicles [3, 7]. FDCs originate from mesenchymal tissue and express specific immunohistochemical markers such as CD21, CD23, CD35, and KiM4 [16]. These positive immunohistochemical markers play a decisive role in the diagnosis of the disease.

The genetic basis of FDCS remains unclear. Andersen and colleagues conducted genomic analysis on 14 FDCS patients and found that most exhibited extensive genomic complexity, primarily characterized by hemizygous losses affecting multiple chromosomes. The study also observed recurrent homozygous deletions involving tumor suppressor genes CDKN2A, RB1, BIRC3, and CYLD, providing new biological insights into the genomic characteristics of FDCS [17]. Sun and others, through microarray analysis, discovered the expression of the epidermal growth factor receptor (EGFR) in FDC sarcomas, with immunohistochemistry confirming moderate to strong expression of EGFR in seven out of eight FDCS cases (88%), suggesting a potential therapeutic target for some surgically challenging cases [18]. Go and colleagues identified the BRAF V600E mutation, a potential drug‐targetable gain‐of‐function mutation [19]. Starr and others discovered pathogenic loss‐of‐function mutations in PTEN (nonsense) and TP53 (missense), along with a novel missense mutation in RET [20]. These genetic alterations expand our understanding of the genomic landscape of FDCS.

The current case demonstrated chest pain in the local mass with the enlargement of tumor size. The patient underwent a CT‐guided fine‐needle biopsy initially. Yet since the lesion was atypical, we did not give a definitive diagnosis. We advised the patient and the clinician to resect the entire tumor for further examination. Complete surgical resection of mediastinal FDCS was performed to clarify the diagnosis and also for the preferred treatment. The disease was eventually diagnosed as FDCS with the assistance of pathological diagnosis and immunohistochemical examination. The patient then received 27 rounds of local radiotherapy without chemotherapy. So far, the patient is still alive. And there is no evidence of recurrence and metastasis to date.

In conclusion, we have described a rare case of extranodal FDCS located in the mediastinum. We should enhance vigilance against the disease and avoid misdiagnosis in the clinic. FDCS should be a consideration when we encounter spindle cell lesions in the mediastinum. The immunohistochemical markers CD21, CD23, and CD35 were very useful in assisting with diagnosis.

## Author Contributions

Data collection and manuscript drafting were undertaken by Qingxia Xu and Chong Zhang. Yang Ma collected and assembled data analysis. Longquan Xiang verified the authenticity of all the raw data. All authors have reviewed and consented to the final manuscript.

## Funding

This work was supported by the Key R&D Program of Jining, 2023YXNS222, and the Natural Science Foundation of Shandong Province, ZR2014HP014.

## Conflicts of Interest

The authors declare no conflicts of interest.

## Data Availability

The datasets utilized and examined in the current study can be provided upon request from the corresponding author.
